# Clinical neuropsychological characteristics of bipolar disorder, with a focus on cognitive and linguistic pattern: a conceptual analysis

**DOI:** 10.12688/f1000research.141599.1

**Published:** 2023-09-27

**Authors:** Evgenia Gkintoni

**Affiliations:** 1 Department of Medicine, University of Patras, Patras, Greece

**Keywords:** Neuropsychology, Bipolar Disorder, Mania, Depression

## Abstract

Neuropsychology is an academic discipline that investigates the intricate interplay between the brain, mind, and behavior. It accomplishes this by examining the underlying structure and activities of the brain, with a particular focus on psychological phenomena such as language, motivation, memory, attention, thinking, consciousness, learning, and efficacy. The assessment of neuropsychological changes in individuals diagnosed with bipolar disorder has received limited attention in comparison to other psychiatric conditions, such as schizophrenia, for instance. Nevertheless, there has been a growing interest in the etiological implications, therapies, preventions, and prognostic factors related to social competence and the quality of life of patients. The objective of this review is to compile and analyze the existing research conducted thus far on the association between cognitive abnormalities and bipolar disorder. This study has examined research conducted across many stages of the condition, including depression and mania. Additionally, it has explored comparative studies involving people with schizophrenia, as well as the potential impact of psychopharmaceutical interventions.

## Introduction

Neuropsychology is the field of study that investigates the intricate link between the brain, mind, and behavior. This inquiry examines the anatomical composition and cognitive mechanisms that serve as the foundation for complex mental activities, including but not limited to consciousness, attention, learning, memory, reasoning, language, motivation, and affectivity ( García & García 2006). The clinical domain of neuropsychology is widely acknowledged as a fundamental field seamlessly incorporated into the flourishing realm of neuroscience and the broader cognitive sciences ( Gkintoni & Dimakos, 2022). Recent models in the field of clinical neuropsychology aim to elucidate cognitive impairments through connectionist theories. These hypotheses propose a novel conceptual framework for understanding brain processes and cognitive functions, drawing upon brain maps of information processing. According to Beaumont *et al.* (1996), this paradigm posits that examining brain processing may elucidate psychiatric problems.

In bipolar disorder, several writers have investigated cognitive impairments across different disorder phases using several clinical instruments, neuropsychological assessments, and neuroimaging modalities for visualizing brain activity ( Sackeim *et al.*, 1983). Nevertheless, it is worth noting that the field of research on this subject has not garnered substantial attention, nor has it been well assessed compared to other mental conditions, such as schizophrenia ( Martínez-Aran *et al.*, 1999a; Miklowitz, 1992).

In a concise historical analysis, it is worth mentioning that early neuropsychological investigations on bipolar disorder indicated the presence of distinct right hemisphere dysfunction. This conclusion was drawn from observations of impaired visuospatial abilities in individuals with bipolar disorder and findings that their intelligence quotient (IQ) scores were comparatively lower on the manipulative scale than on the verbal scale ( Dalby and Williams, 1986). The hypothesis, proposed initially by Flor-Henry and Yeudal in the late 1970s, has undergone various revisions. For instance, Kluger and Goldberg conducted a meta-analysis in 1990, revealing that affective patients' characteristics were more similar to those with diffuse bilateral brain injury rather than individuals with lesions in the right hemisphere ( Kluger and Goldberg, 1990). Furthermore, the previous study conducted by Bearden *et al.* (2001) highlights that individuals with bipolar disorder typically do not exhibit visuospatial impairments in straightforward tasks during affective episodes. However, these deficits arise during more intricate tasks involving memory, nonverbal conceptualization, and abstract reasoning abilities. As a result of this observation, they contend that the cognitive profile does not align with localized impairment alone in the right hemisphere but rather with the interplay of multiple integrated neuroanatomical systems, encompassing frontal-executive processes, attention, and memory ( Gkintoni *et al.*, 2022a).

Conversely, there has been a growing interest in examining cognitive impairments in individuals with bipolar disorder in recent years ( Martinez-Aran *et al.*, 2005). This interest has been fueled, at least partly, by studies aiming to identify potential similarities or differences in the neurocognitive functioning of patients with bipolar disorder compared to those with schizophrenia, often used as comparison groups in research studies ( Gkintoni *et al.*, 2017). Despite conflicting findings and a lack of consistency in the samples utilized, these comparative studies have generated hypotheses regarding the shared and distinct neuropsychological and neurobiological foundations of both disorders. Additionally, they have contributed to the development of conceptual and methodological frameworks within various domains of neuropsychiatry.

The earliest comparative investigations have generated a growing interest in the involvement of cognitive functions in bipolar disorder regarding its etiological implications and potential treatments for patients. Notably, impairments in neurocognitive functioning can be highly significant in their ability to serve as markers of functional prognosis ( Antonopoulou *et al.*, 2021,2022). They can predict various outcomes, such as adjustment, social competence, and quality of life in individuals affected by these deficits ( Austin *et al.*, 2001).

Hence, there is a recognized need to conduct further research to define the neuropsychological profiles of individuals in both the acute and clinical remission stages of bipolar illness. This will enable the formulation of epistemic and intervention theories. Research conducted on individuals with bipolar disorder during non-asymptomatic phases offers the potential to uncover cognitive characteristics.

Bipolar disorder is a psychiatric disorder distinguished by alternating episodes of mania and depression. The clinical manifestations of bipolar disorder are widely recognized, but there is an increasing scholarly focus on comprehending the cognitive aspects of this condition. Neuropsychological functioning pertains to the cognitive capacities and mechanisms linked to the brain's functioning ( Gkintoni *et al.*, 2023). Numerous research has been conducted to examine the neuropsychological functioning of individuals diagnosed with bipolar disorder, focusing on their cognitive abilities during periods of euthymia ( *i.e.*, stable mood states). Torres *et al.* (2007) conducted a meta-analysis to investigate the neuropsychological performance of persons diagnosed with euthymic bipolar illness compared to a control group of healthy individuals. The research revealed notable disparities of medium-to-large effect sizes in attention/processing speed, episodic memory, and executive functioning between the sick and control groups. The results of this study indicate that patients diagnosed with bipolar disorder may exhibit cognitive impairments in these categories. Martínez-Arán *et al.* (2005) conducted an independent study to examine and compare the cognitive abilities of patients with bipolar exhibiting high-functioning and low-functioning characteristics concerning a control group of individuals without bipolar disorder. The study found that patients with bipolar illness have worse cognitive function than non-patients. Verbal memory and executive functioning were impaired in cognitively impaired people.

Bora *et al.* (2009) did a meta-analysis to examine cognitive deficits in affective psychoses such as bipolar illness and major depressive disorder. The study found that patients with bipolar disorder had similar attentional processing, learning, and memory impairments to patients with euthymic bipolar disorder. Cognitive impairments may be widespread in emotional psychoses, according to this study. Additionally, several researchers have examined specific cognitive domains in patients with bipolar disorder. Clark *et al.* (2002) found attention abnormalities in patients with bipolar disorder. This suggests that these inadequacies may indicate disorder vulnerability. Reinke *et al.* (2013) examined language-processing brain areas and verbal memory in patients with euthymic bipolar illness. The study found changes in intrinsic functional connectivity across brain regions connected to language, suggesting a relationship between language deficiencies and verbal memory delays in bipolar disorder. It is essential to understand that bipolar disorder might cause cognitive impairments after euthymia. Cognitive impairments may occur during manic and depressive periods, according to research. In depressive episodes, patients with bipolar disorder have abnormalities in reward processing, short-term spatial memory recall, and sensitivity to negative feedback, according to Roiser *et al.* (2009). This study suggests that the impairments are caused by the disease rather than mood-stabilizing medicines. Data suggests that bipolar disorder may impair attention, processing speed, memory, executive functioning, sustained attention, and language. The illness's euthymic and symptomatic phases may reveal these shortcomings. Further research is needed to understand the genesis and therapeutic implications of bipolar disorder's cognitive features.

## Neurobiological pattern in bipolar disorder

Bipolar disorder is a complex mental illness that causes mania and sadness. The neurobiology of bipolar disorder has been studied extensively to determine its processes and biomarkers. The neurological correlates of bipolar disorder have been shown *via* neuroimaging. Strakowski *et al.* (2012) and ( 2004) found anomalies in brain areas and networks involved in emotional regulation, cognitive processing, and reward systems. The pathophysiology of bipolar disorder has been linked to alterations in multiple brain regions, such as the prefrontal cortex, amygdala, striatum, thalamus, and limbic circuits ( Strakowski *et al.*, 2012; Strakowski *et al.*, 2004; DelBello *et al.*, 2004). The investigation of bipolar disorder has explicitly focused on the prefrontal cortex, particularly the ventromedial prefrontal cortex (vmPFC). The presence of dysfunction within the vmPFC and its interconnections with other cerebral regions, including the amygdala and striatum, has been linked to the occurrence of emotional dysregulation and mood instability ( Strakowski *et al.*, 2012; Abé *et al.*, 2021).

Disturbances may influence the occurrence and advancement of bipolar disorder in the connection and regulation of the aforementioned brain regions ( Strakowski *et al.*, 2012; DelBello *et al.*, 2004). The neurobiological characteristics of bipolar disorder are also influenced to a great extent by genetic factors. Previous research has successfully discovered genetic risk factors linked to changes in the brain's structural and functional aspects, specifically in the vmPFC ( Abé *et al.*, 2021). The neurological mechanisms underlying bipolar illness are further influenced by the interaction between hereditary predisposition and environmental circumstances ( Abé *et al.*, 2021). Cognitive deficiencies are frequently found in patients diagnosed with bipolar disorder, and research in neurobiology has yielded valuable insights into the brain underpinnings of these impairments. Cognitive dysfunction in bipolar disorder has been linked to abnormalities in brain regions implicated in cognitive functions, including the prefrontal cortex, temporal lobe, and cerebellum ( Strakowski *et al.*, 2004; Simonsen *et al.*, 2008). The comprehension of the neurobiological underpinnings of cognitive deficits might provide valuable insights for designing and implementing specific therapies to enhance cognitive performance in individuals diagnosed with bipolar illness.

In summary, findings from neurobiological investigations on bipolar disease have shown deviations in brain morphology, connectivity, and activity, specifically within areas implicated in emotional regulation, cognitive functioning, and reward mechanisms ( Gkintoni *et al.*, 2021). The involvement of the prefrontal cortex, limbic circuits, and their interplay has been identified as playing a role in the pathophysiology of bipolar disorder ( Coffman *et al.*, 1990). The neurobiological patterns found in this illness are additionally influenced by genetic variables and their interplay with environmental influences. Ongoing investigation in this domain is necessary to enhance our comprehension of the fundamental mechanisms further and facilitate the development of more efficacious interventions for persons diagnosed with bipolar illness.

## Neuropsychological findings during affective episodes

Typically, within the context of therapeutic practice, it is commonly noted that these patients present with cognitive challenges. Furthermore, many of these individuals have exhibited cognitive decline and deterioration throughout the acute stages ( Sweeney *et al.*, 2000). According to Martínez-Aran *et al.* (2007), there have been observed variations in the fluency of thought and speech, impairments in learning and memory, and modifications in associative patterns and attentional processes during both depressed and manic episodes. Challenges have also been noted in activities that necessitate sequential processing and evaluate cognitive abilities at a higher level, such as abstraction and adaptability ( Bearden *et al.*, 2001).

Certain researchers have observed comparable impairments in both types of affective episodes ( Bulbena and Berrios, 1993) and suggest the presence of a widespread cognitive dysfunction that may be linked to a shared pathophysiological mechanism involving multiple neuronal regions, including the dorsolateral prefrontal cortex. This brain region maintains numerous connections with cortical systems involved in information processing. However, alternative research suggests that individuals with bipolar I disorder exhibit distinct cognitive profiles during episodes of mania and depression ( Sweeney *et al.*, 2000). These differences are primarily observed in tasks that require the emotional processing of stimuli. Murphy *et al.* (1999) suggest that the observed cognitive variations could be associated with divergent activation patterns within the subgenual area of the corpus callosum. Drevets *et al.* (1997) found that this brain area is overactive during manic episodes and underactive during depressive periods. Thus, the ventromedial prefrontal cortex, which is highly connected to the limbic system, may be involved in these cognitive processes. Research shows that individuals experiencing a manic episode often have trouble focusing. This deficiency is connected to diminished prefrontal and hippocampal cortical volume, according to Sax *et al.* in 1999. The deficit seen in mania is often attributed to attention impairment ( Clark *et al.*, 2001). Distraction in healthcare situations is also important. Subsequent investigations have revealed a significant decline in executive functioning, as evidenced by deficiencies in cognitive tasks such as the Tower of London ( Sweeney *et al.*, 2000) and the Stroop Color-Word Stroop test ( Golden *et al.*, 2002; McGrath *et al.*, 1997). In addition, researchers have also noted impairments in spatial working memory ( Sweeney *et al.*, 2000). Several studies have examined the cognitive deficits associated with manic episodes. For instance, Sweeney *et al.* (2000) found impairments in various aspects of memory, while Clark *et al.* (2001) identified deficits specifically in verbal learning. McGrath (2001) posit that some scholars have proposed a potential link between the observed memory impairments during this phase of the disorder and altered patterns of verbal association ( Fleck *et al.*, 2003).

In the realm of language, particularly concerning verbal fluency, while certain studies have failed to identify impairments during manic episodes ( Calev *et al.*, 1989), a recent investigation ( Lebowitz *et al.*, 2001) has demonstrated that individuals with a more remarkable history of manic episodes exhibit a substantial number of errors in both phonological and semantic fluency tasks.

In a study conducted by Bearden *et al.* (2001), a limited number of patients with bipolar disorder were examined across various phases of the disorder. The findings revealed that individuals with bipolar disorder exhibited a deficit in verbal learning ability while in a manic state compared to their performance during the remission phase. However, no significant differences were observed in short-term free recall ability across different mood states. These results led the authors to propose that the experience of mania may specifically impact complex processing or memory functions while leaving more straightforward cognitive tasks unaffected.

In the context of examining symptomatic episodes of schizophrenia and mania, certain researchers ( Morice, 1990) have observed that there are no discernible distinctions in the neurocognitive impairment profiles of patients with schizophrenia and bipolar disorder. This observation is particularly evident in tasks that evaluate executive function, such as the Wisconsin Card Sorting Test. Consequently, it can be inferred that some of the deficits identified in these patients are not exclusive to either diagnostic category. However, according to Goldberg *et al.* (1993), there is evidence of more pronounced dysfunction in the frontal region among individuals diagnosed with schizophrenia. Additionally, Raust *et al.* (2014) propose the possibility of temporary global brain dysfunction in individuals experiencing mania and a more enduring prefrontal dysfunction in those with schizophrenia.

During episodes of depression, individuals diagnosed with bipolar disorder have impairments in memory, particularly in the episodic aspect. This observation, as reported by Sweeney *et al.* (2000), indicates a resemblance between patients with bipolar and patients with unipolar depression, implying the presence of temporal lobe dysfunction in both cohorts. Nevertheless, Borkowska and Rybakowski (2001) conducted a study that revealed distinct cognitive profiles between individuals with bipolar and unipolar depression. Specifically, individuals with bipolar depression had poorer performance on tasks assessing some components of frontal lobe functions. The heightened cognitive impairment observed in individuals with bipolar depression and its correlation with frontal brain activity may indicate potential variations in the underlying causes compared to unipolar affective illness.

By contrast, a subsequent investigation conducted by Donnelly, Murphy, Goodwin, and Waldman (1982) revealed that the patients mentioned above exhibited diminished cognitive abilities, as indicated by lower IQ scores, during periods of depression compared to their euthymic phase. This decline in IQ scores persisted even when accounting for practice effects, as the reduction was observed again during subsequent depressive episodes.

One of the explanations proposed regarding the cognitive impairments observed in bipolar disorder during acute phases is presented by Albus *et al.* (1996). They argue that these deficits result from the presence of psychotic symptoms and their impact on neuropsychological functioning rather than being solely attributed to the clinical diagnosis of bipolar disorder. However, it is worth noting that subsequent research, as indicated by Mojtabai *et al.* (2000), has not confirmed this assertion. Furthermore, Micklowitz *et al.* (1992) conducted additional research that indicates individuals diagnosed with bipolar disorder who exhibit symptoms of psychosis are prone to have a more severe and persistent illness trajectory. This finding suggests that these patients may therefore encounter more pronounced cognitive decline as time progresses. Nevertheless, there remains a dearth of comprehensive research investigating the precise impact of psychotic symptoms on cognitive functioning within bipolar disorder.

## Neuropsychological findings in euthymia

In recent years, there has been an accumulation of empirical data indicating the presence of cognitive impairments in many individuals diagnosed with bipolar disorder during periods of remission or euthymic states ( Taylor & Vaidya, 2005). The results of this study indicate that specific cognitive impairments are not limited to affective episodes or dependent on the individual's current condition but persist even after the resolution of such events. This suggests that individuals with bipolar disorder do not fully recover between episodes. This statement contradicts Kraepelin's original proposition on manic-depressive psychosis, as he posited that it does not exhibit a decline in functioning following the acute phase, in contrast to schizophrenia ( Ferrier and Thompson, 2002). The classical understanding of bipolar disorder was primarily embraced within psychiatry, even without comprehensive empirical investigations, and has persisted throughout the years.

However, there is an ongoing debate in neuropsychology regarding the cognitive impairments present during the acute phases of a condition and if these impairments continue in a state of clinical remission ( McGrath 2001). One could posit that most of these deficits resolve during periods of euthymia, with only a subset appearing to endure ( Altshuler, 1993). It is not commonly observed that there is a pervasive intellectual deficit, but rather indications of constraints in certain cognitive functions or information processing strategies ( Bearden *et al.*, 2001).

Nevertheless, there is a lack of clarity in the medical literature regarding whether the deficits observed can be attributed to a decline in cognitive function that occurs as the disorder progresses or if they are pre-existing in individuals before the onset of clinical episodes of bipolar disorder. This ambiguity suggests the potential presence of trait markers rather than cognitive dysfunctions dependent on the clinical state. The literature contains several research ( Sigurdsson *et al.*, 1999) that examine the correlation between bipolar disorder and a history of delayed motor, social, and language development in individuals. Nevertheless, the limited body of research on this matter hinders the ability to ascertain whether cognitive dysfunction indicates susceptibility to bipolar disorder.

Comparative investigations conducted on individuals in the asymptomatic phase of bipolar illness and schizophrenia have failed to yield definitive findings about the cognitive profiles exhibited by individuals with both disorders during periods of remission. Morice (1990) reported no statistically significant distinctions in neurocognitive impairment profiles. However, subsequent research conducted by Hawkins *et al.* (1997) revealed that patients with euthymic bipolar exhibited poorer performance in attentional tests assessing visuomotor speed (specifically, Digit-Symbol and Trail Making Test) compared to stabilized individuals with schizophrenia. Nevertheless, the prevailing evidence consistently indicates the presence of notable cognitive impairment in individuals diagnosed with schizophrenia across a range of cognitive domains. Specifically, studies have revealed deficits in psychomotor speed, attention, and working memory ( Elvevag and Goldberg, 2000), executive functions ( Krabbendam *et al.*, 2000), and verbal function ( Gruzelier *et al.*, 1988). Notably, these cognitive deficits are more pronounced in patients with schizophrenia than in individuals with bipolar and unipolar affective disorders. The results mentioned above align with traditional nosological concepts, which posited that bipolar disorder, in contrast to schizophrenia, does not exhibit a pervasive cognitive deficit or impairment that is unrelated to illness episodes or the premorbid state ( Miller, 1975). However, it is worth noting that this perspective has been subject to reevaluation in recent times.

The abovementioned questions about stability and the selectivity and specificity of cognitive deficits in bipolar disorder continue to lack clarity. The current situation can be attributed, at least in part, to the limited amount of scientific research conducted on neuropsychological assessment in euthymic individuals with bipolar disorder ( Miller, 1975). Furthermore, the existing body of literature generally lacks definitive findings regarding cognitive impairments in patients with bipolar I disorder during the euthymic phase ( Hawkins *et al.*, 1997).

The observed variations in the outcomes are attributed to the presence of errors or methodological inconsistencies across most studies. These errors include the utilization of diverse and occasionally ambiguous remission criteria, diagnostic heterogeneity, limited sample sizes, inadequate control over the impact of pharmacological treatment and subclinical symptoms, and the influence of practice ( Martínez-Aran *et al.*, 1999b). It is essential to highlight the significant lack of longitudinal studies incorporating cognitive assessments before and after the onset of the disorder, as well as during periods of illness and clinical remission. These studies would provide valuable insights into whether alterations in intellectual function are attributable to clinical status, genetic predisposition, or treatment conditions ( Stoll *et al.*, 1996).

The final variable under consideration, medicine, holds significance due to its impact on psychological functions, as evidenced by studies on the administration of psychopharmacological therapies such as lithium ( Ananth *et al.*, 1987). These medications have been found to induce memory impairments and psychomotor deceleration potentially. Nevertheless, it is frequently seen that patients do not exhibit these deficiencies ( Honig *et al.*, 1999). A study conducted by Kessing (1998) revealed a significant correlation between the frequency of episodes and lithium medication and two of the five assessments of overall cognitive performance. In a study conducted by Lund *et al.* ( Bearden *et al.*, 2001), it was discovered that individuals with bipolar disorder who received long-term treatment with lithium exhibited average performance levels on attention and memory tasks compared to the general population ( Shaw *et al.*, 1987). Similarly, Engelsmann, Katz, Ghadirian, and Schachter ( Engelsmann *et al.*, 1988) observed that the memory scores of patients with bipolar treated with lithium, both in the short and long term, remained consistently stable over six years.

According to Bearden *et al.* (2001), concentration difficulties may arise due to using alternative mood stabilizers, such as carbamazepine or valproate. Nevertheless, Thompson *et al.* (2006) have presented research suggesting that individuals with epilepsy may improve their attention span when undergoing carbamazepine treatment compared to alternative anticonvulsant medications.

According to Martinez-Aran *et al.* (2002), tricyclic antidepressants have been found to enhance cognitive function, but they may have a negative impact on memory. Similarly, long-term antipsychotic medication has been associated with improved overall performance. Nevertheless, when administered in high quantities, it has been found to potentially result in decreased performance on verbal memory or attention assessments ( Cassens *et al.*, 1990). Regarding this matter, most of the research undertaken on antipsychotics, explicitly focusing on individuals diagnosed with schizophrenia, has reached the consensus that the cognitive impairments identified in these patients are primarily attributable to the disorder itself rather than the treatment with antipsychotic medication.

The collective results indicate that although medication may result in a certain level of cognitive deceleration, the neuropsychological impairments observed in individuals with bipolar disorder do not seem to be a consequence of pharmaceutical intervention ( Bearden *et al.*, 2001). Nevertheless, it is imperative to acknowledge that there remains a dearth of knowledge regarding the impact of medication on cognitive abilities in individuals with bipolar disorder, given the limited number of studies conducted within this specific population. Furthermore, it is imperative to consider the challenges that arise when evaluating the impact of psychotropic drugs on patients who are frequently prescribed many medications and may even get varying dosages over a period.

Furthermore, additional findings derived from some research studies suggest that the observed effect is more likely attributable to the progression and development of the condition rather than the existence of endophenotypes or markers on a neuropsychological level. Therefore, previous research has proposed that individuals with recurrent or severe mood disorders may experience deficits in neuropsychological functioning during the euthymic phase ( Zarate *et al.*, 2000; McKay *et al.*, 1995). Additionally, the number of episodes, particularly those characterized by depression, may impact these impairments, as indicated by Kessing *et al.*'s study ( 1998). The study by Tham *et al.* (1997) revealed a notable correlation between decreased cognitive functioning and increased occurrences of relapses or episodes of mania or depression, as well as a greater frequency of hospitalizations compared to individuals with normal cognitive functioning. Denicoff *et al.* (1999) observed that individuals with a more severe course of illness, characterized by a higher number of episodes and hospitalizations and a longer duration of sickness, exhibited poorer performance in memory, attention, and abstraction tasks. Similarly, previous researchers ( Cavanagh *et al.*, 2002) have likewise documented a potential collective adverse impact on the verbal learning and memory systems, as well as the frontal or executive system, concerning the length of the disorder and occurrences of manic and depressive episodes.

Hence, there has been a proposition suggesting that affective episodes could potentially result in brain tissue damage or lesions, referred to as a psychic imprint or scar. This proposition implies that the persistent cognitive deficits observed during the euthymic phase may serve as a functional manifestation of such damage, particularly concerning learning and memory functions ( Altshuler, 1993). Nevertheless, the idea suggesting a gradual deterioration in cognitive functioning from the premorbid state concerning the trajectory of bipolar disorder lacks empirical validity, as indicated by long-term follow-up studies ( Dhingra and Rabins, 1991). The extent to which the deficiencies reported in previous investigations ( Savard *et al.*, 1980) can be attributed to the senior age of the participants tested rather than the chronicity or severity of the disorder remains uncertain. Moreover, it is conceivable that the existence of chronicity and cognitive impairment could indicate the disorder's severity. Patients with bipolar illness with reduced cognitive ability may have more episodes. Thus, chronicity may signal cognitive deficits and the condition rather than causing cognitive decline ( Tohen *et al.*, 1990; Tohen, 2000). However, more research is needed to prove this concept.

The sustained cognitive deficiencies experienced by patients with bipolar illness during the euthymic phase have caused much debate. Recent research has focused on cognitive functions, particularly memory and executive function. Due to their frequent association with maladaptive tendencies, these domains have drawn examination. Ferrier and Thompson (2002) found lasting memory and executive function deficits alone or in combination.

## Learning and memory

Multiple studies have provided evidence of a decrease in cognitive abilities, specifically in the domains of learning and memory. Specifically, the impairment has been observed in verbal or explicit declarative memory, as assessed by the California Verbal Learning Test (CVLT) ( Cavanagh *et al.*, 2002; Delis *et al.*, 1987). Hence, a decrease in learning and memory of words was reported in euthymic individuals with bipolar disorder compared to control subjects, specifically regarding accessible and directed recall ( Atre-Vaidya & Hussain, 1999; van Gorp *et al.*, 1999).

The findings presented in this study have been independently verified by other researchers ( Ferrier *et al.*, 1999), who propose that specific memory impairments continue to exist even during remission in individuals with bipolar disorder ( Thompson *et al.*, 2001; Thompson, 2007). Nevertheless, there remains uncertainty regarding the potential impact of subclinical depressive-type symptomatology on this dysfunction, which could be observed in individuals with euthymic bipolar disorder ( Ferrier *et al.*, 1999). Additionally, it is unclear whether various factors associated with the progression of the disorder, such as the frequency of depressive episodes, the duration of bipolar disorder ( Van Gorp *et al.*, 1998), or the number of manic episodes ( Cavanagh *et al.*, 2002), may also contribute to this dysfunction.

Therefore, the current findings do not provide sufficient evidence to determine whether the observed learning and verbal memory impairments mentioned earlier can be considered as inherent cognitive traits of these patients, as indicated by Gourovitch *et al.* (1999) in their investigation of monozygotic twins who differ in their bipolar disorder status. This study compared twins who were affected, unaffected, and those without the disorder. The results of this study suggest that genetic factors associated with the disorder may contribute to subtle alterations in overall memory or retrieval function rather than being directly implicated in its initiation and advancement.

## Executive function

The cognitive domain, referred to as executive function, holds significance due to its inclusion of a range of advanced cognitive processes, including but not limited to planning, decision-making, problem-solving, cognitive flexibility, and inhibitory control ( Gkintoni *et al.*, 2022b; Halkiopoulos *et al.*, 2022). Extensive research has been conducted on the clinical neuropsychological characteristics of bipolar disorder, with a particular focus on examining executive function. In their study, Bora and Pantelis (2015) undertook a meta-analysis intending to examine and contrast the cognitive impairment observed in individuals who have undergone their initial episode of bipolar disorder (FEBP), the inaugural episode of schizophrenia (FES), and a control group comprising individuals without any psychiatric disorders. The study found that patients with Frontal Epilepsy-Related Brain Pathology (FEBP) had severe cognitive deficits, notably in executive tasks. In numerous cognitive domains, including executive skills, functional electrical stimulation (FES) was inferior to functional electrical brain stimulation (FEBP). This study suggests that patients with bipolar disorder and schizophrenia have executive function issues. Bora and Pantelis (2015) found that patients with bipolar disorder experiencing their first episode have impairment levels between schizophrenia and those without psychiatric disorders.

In addition to meta-analyses, Sparding *et al.* (2021) did a longitudinal investigation of the cognitive functioning of patients with bipolar illness. The study found a substantial difference in executive functioning speed and accuracy between patients with bipolar I and II. The findings of Sparding *et al.* (2021) indicate the potential existence of variations in executive function between individuals diagnosed with bipolar I disorder and those diagnosed with bipolar II disorder. The examination of the relationship between executive function and everyday functioning in individuals with bipolar disorder has also been conducted. The study conducted by Depp *et al.* (2012) employed a meta-analytic approach to investigate the association between cognitive abilities and everyday functioning in individuals diagnosed with bipolar disorder. The research revealed that individuals diagnosed with bipolar disorder frequently experience neurocognitive impairments, particularly in executive function, significantly impacting their ability to function effectively. The level of correlation between cognitive ability, specifically executive function, and everyday functioning was found to be comparable to the correlation observed in individuals with schizophrenia ( Depp *et al.*, 2012).

While it is frequently observed that patients with bipolar in a euthymic state exhibit abnormalities in executive function, significant disagreement exists regarding the precise degree to which impaired executive functioning is specifically linked to bipolar disorder ( Rubinsztein *et al.*, 2000).

According to a study conducted by Ferrier and Thompson (2002), it has been noted that individuals with bipolar disorder who are in a euthymic state demonstrate inferior performance on tasks related to executive function, notably the Wisconsin Card Sorting Test ( Graham Beaumont, 1981; Heaton, 1981). This phenomenon is notably apparent when examining the number of accomplished categories and perseverative errors in individuals diagnosed with bipolar disorder compared to a control group of individuals without the disorder who are in good health.

However, several studies have been unable to detect any deficiencies in different tests assessing frontal function, such as the Trail Making Test (TMT) and the FAS, as documented by Van Gorp *et al.* (1998) and other scholars ( Bauwens *et al.*, 1991; Cavanagh *et al.* 2002). Nevertheless, it is imperative to acknowledge that instances that deviate from this prevailing pattern exist. An example of a study conducted by Ferrier *et al.* (1999) involved observing changes in the TMT, FAS, and Tower of London tests among patients in remission. The abovementioned modifications became apparent solely after accounting for age, premorbid IQ, and subclinical depressive symptoms.

However, certain studies have proposed that executive deficits may not manifest initially at the onset of the disorder but rather emerge and progress throughout the illness. The chronic nature of the disorder can potentially impact these deficits, resulting in confusion and challenges in differentiating between executive deficits and the duration of the disorder ( Martinez-Aran *et al.*, 2002). This observation suggests that the maintenance of clinical remission and the prevention of relapse may function to hinder further decline and potentially promote progress.

In general, the existing body of literature consistently suggests that individuals diagnosed with bipolar disorder may exhibit impairments in executive function. The deficits mentioned above are evident during both the symptomatic and euthymic phases of the disorder, and they play a role in causing functional impairment. Nevertheless, it is imperative to acknowledge that the presence of executive function impairments in individuals with bipolar disorder is not consistently observed across all studies. Therefore, additional research is warranted to understand better the underlying mechanisms and potential therapeutic implications associated with these neuropsychological traits.

## Attention

Attention is a core cognitive function that plays a fundamental role in many higher-order cognitive processes, from memory to executive functions. Attentional deficits in bipolar disorder (BD) can substantially impact various aspects of daily functioning, the trajectory of the illness, and its overall prognosis. An in-depth examination of attention as a prominent factor in the clinical neuropsychological manifestations of bipolar disorder pertains to deficits in attention observed throughout various phases ( Wilder-Willis *et al.*, 2001).

Prior studies have demonstrated that many individuals diagnosed with bipolar disorder exhibit deficits in their attentional system during acute episodes. The manifestation of this dysfunction is notably apparent in assessments of selective attention, such as the Stroop Color-Word test, as well as sustained attention, exemplified by the continuous performance test. Nevertheless, it seems that these attentional deficits are less prevalent among individuals diagnosed with bipolar disorder who are experiencing a euthymic state. The discovery was reported by Quraishi and Frangou in 2002. Many feel that selective attentional deficiencies rely on a person's state. Van Gorp *et al.* (1998) found that selective attention is unaffected by euthymia.

However, research has shown that patients with bipolar illness may have chronic concentration issues ( Clark *et al.*, 2002). This is particularly true for people with subjective cognitive difficulties ( Martinez-Aran *et al.*, 2002). These issues may be more likely in people with more recurrent depression or longer-term bipolar disorder ( Clark *et al.*, 2002).

In some circumstances, early attentional mechanisms may impede psychomotor slowing. Cognitive impairment can be reversed, especially after depressive episodes end. This illness can also cause depression-like symptoms including intrusive rumination or mania-like symptoms such as focus issues. Psychomotor slowing is linked to motivation and pharmacological therapies like lithium or antipsychotics ( Martinez-Aran *et al.*, 2002; Shaw *et al.*, 1987). This implies that the manifestation of psychomotor slowing is contingent upon the individual's current state ( Paradiso *et al.*, 1997). However, it is worth noting that specific authors have observed a consistent impairment in psychomotor speed among patients with bipolar, even during periods of euthymia ( Hawkins *et al.*, 1997).

In summary, examining attention is a pivotal factor in comprehending the neuropsychological terrain associated with bipolar disorder. The improvement of attentional deficits through addressing and rehabilitating them has been shown to substantially impact the overall quality of life and functional outcomes of individuals diagnosed with BD.

## Language

Comprehending the neuropsychological and language characteristics of bipolar disorder can be advantageous in the clinical context for diagnosis and intervention. Linguistic patterns provide a concrete and observable collection of actions that assist physicians in assessing an individual's position within their mood cycle and the intensity of their symptoms at that time. Integrating this approach with a comprehensive neuropsychological evaluation yields an extensive comprehension of the individual's condition ( Graham Beaumont, 1981). During episodes of mania, individuals may display symptoms of pressurized speech characterized by quick, excessive, and urgent talking patterns. Individuals with this communication style may exhibit challenges in being interrupted and may demonstrate a tendency to transition rapidly between different subjects. Individuals diagnosed with bipolar disorder, particularly during episodes of mania, may display tangential speech, characterized by a tendency to deviate from the central topic without returning to it, or circumstantial speech, which includes extraneous details and a prolonged approach to conveying the main message. Specific individuals diagnosed with bipolar disorder (BD) have reported experiencing heightened levels of verbal creativity, whether in hypomania or mania. The individuals in question may opt to employ a more intricate lexicon, establish distinctive language associations, or use poetic or metaphorical discourse. During the depression, individuals may experience a decrease in the frequency of their speech and an increase in its monotony. The individual may exhibit diminished verbal expression and employ a restricted lexicon, indicative of their depressive state. During episodes of intense mania or depression, individuals may experience challenges in comprehending a person's words due to the disorganization of their ideas. This may be incoherent or characterized by "word salad" speech patterns.

Regarding language, disruptions in communication among patients with bipolar who are generally stable appear to be associated with worse performance in tasks that require concept formulation and verbal fluency. On the other hand, in patients with schizophrenia, these disruptions are more likely to be connected to attention and working memory processes. Nevertheless, there exist disparities in the findings of the studies mentioned above. Van Gorp *et al.* (1998) reported insignificant disparities in verbal fluency between individuals diagnosed with bipolar disorder and those without the condition. Conversely, Ferrier *et al.* (1999) observed substandard performance in fluency tasks among individuals with bipolar disorder who were in a euthymic state. Moreover, it has been postulated that these disparities may be associated with depressed subsyndromal symptomatology ( Martínez-Aran *et al.*, 1999a).

Concerning this matter, it is worth noting that while patients are commonly characterized as asymptomatic or euthymic in research studies, many exhibit subsyndromal fluctuations that impact their overall functioning. These fluctuations appear to be associated with cognitive dysfunctions, as discussed in the literature ( Ferrier and Thompson, 2002), and with suboptimal psychosocial adjustment ( Bauwens *et al.*, 1991). Hence, there is a significant scholarly interest in investigating the occurrence and characteristics of subclinical symptoms in individuals with euthymic bipolar disorder and quantifying them through clinical symptom assessment scales (specifically for depression and mania). This research aims to explore the impact of these symptoms on patients' cognitive abilities, as assessed using neuropsychological testing. In conclusion, the results of the studies suggest lasting cognitive deficits in a specific group of individuals with bipolar disorder during periods of mood stability. These impairments are most observed in the domains of memory and executive function, which may suggest the presence of inherent characteristics rather than temporary alterations. However, these findings sometimes appear primarily linked to clinical and pharmacological factors. Hence, their research entails examining and regulating several factors that could impact the occurrence of these impairments, including subclinical or subsyndromal symptomatology, pharmaceutical intervention, and the trajectory and progression of the condition.

## Discussion and conclusions

Bipolar disorder is distinguished by alternating episodes of mania and depression. The clinical manifestations of bipolar disorder have been extensively examined, prompting a surge of scholarly attention towards comprehending the neuropsychological aspects of this condition. Neuropsychological functioning pertains to the cognitive capacities and mechanisms that underpin behavior and mental processes. Numerous studies have been conducted to examine the neuropsychological functioning of individuals diagnosed with bipolar disorder. Torres *et al.* (2007) conducted a meta-analysis to investigate the neuropsychological performance of individuals diagnosed with bipolar disorder who were in a euthymic state characterized by the absence of symptoms. The above research revealed that individuals diagnosed with bipolar disorder exhibited impairments in attention and processing speed, episodic memory, and executive functioning compared to those without the disorder.

The neuropsychological impairments observed in individuals with bipolar disorder have been subject to comparison with those observed in individuals with schizophrenia. The study conducted by Hill *et al.* (2013) investigated the neuropsychological impairments observed in individuals diagnosed with schizophrenia and psychotic bipolar disorder. The research revealed that individuals with bipolar disorder, similar to those with schizophrenia, exhibited persistent cognitive deficits characteristic of enduring traits. Nevertheless, a greater degree of cognitive impairments was consistently observed in individuals diagnosed with bipolar disorder who also experienced psychosis compared to those with bipolar disorder who did not exhibit psychotic symptoms. The present findings indicate that there may exist resemblances in the neuropsychological deficits observed in individuals with bipolar disorder and schizophrenia while also highlighting particular distinctions associated with psychosis. Researchers have also examined the correlation between cognitive impairments and daily functioning in individuals diagnosed with bipolar disorder. Depp *et al.* (2012) conducted a meta-analysis to investigate the correlation between cognitive abilities and everyday functioning in individuals diagnosed with bipolar disorder. The research revealed a significant correlation between cognitive impairments and functional disability in individuals diagnosed with bipolar disorder. The extent to which cognitive impairment affects functioning varies across distinct cognitive domains and functional indicators. The findings mentioned above indicate that cognitive deficits associated with bipolar disorder can substantially impact individuals' day-to-day functioning.

To summarize, investigations into the neuropsychological aspects of bipolar disorder have yielded findings supporting cognitive deficits in attention and processing speed, memory, and executive functioning. These impairments have the potential to manifest in individuals with both euthymic and symptomatic bipolar disorder and can have adverse effects on their psychosocial and daily functioning. The cognitive impairments observed in individuals with bipolar disorder exhibit similarities to those observed in individuals with schizophrenia, although distinctions may exist associated with psychosis. The existing comprehension regarding the stability, selectivity, and specificity of cognitive impairments in individuals with bipolar disorder is constrained due to several methodological inconsistencies. The observed disparities encompass variations in the criteria used to determine remission, diverse diagnostic profiles, limited sample sizes, and insufficient control over the influence of pharmacological treatment, subclinical symptoms, and practice effects.

Additional investigation is required to understand better the fundamental mechanisms and potential factors that may moderate neuropsychological impairments in individuals with bipolar disorder. Further research is necessary to ascertain the neuropsychological characteristics of individuals during the acute and clinical remission phases of bipolar disorder. This will facilitate the formulation of epistemological perspectives and hypotheses for intervention.

This study highlights the notable association between cognitive impairment and bipolar disorder. Despite the extensive corpus of evidence suggesting cognitive impairments in individuals diagnosed with bipolar disorder, a dearth of comprehensive comprehension of the neuropsychological profiles these patients display persists. Acquiring such knowledge is essential to effectively evaluate the intensity of symptoms, assess the effectiveness of treatment, and make predictions about functional outcomes. The impairments, as mentioned above, encompass disturbances in memory, attention, executive functioning, and language, are not limited to affective episodes but endure consistently even during periods of stable mood, suggesting a possible enduring nature to these deficits. However, there remains a dearth of clarity on whether these cognitive impairments indicate bipolar disorder or if they predate the onset of the disorder. Clarifying this matter is of utmost importance to improve diagnostic accuracy and develop more efficacious treatment approaches.

## Data Availability

No data are associated with this article.
