# Supplementary material for: Clinical neuropsychological characteristics of bipolar disorder, with a focus on cognitive and linguistic pattern: a conceptual analysis
Source: F1000Res. 2023 Sep 27;12:1235. [Version 1] doi: 10.12688/f1000research.141599.1 (PMC10905171; doi:10.12688/f1000research.141599.1)
Supplement: Peer review history

# Reviewer report: Reviewer response for version 1

**DOI:** 10.5256/f1000research.155060.r220344

**Published:** 2024-02-08

**Authors:** Elias Kourkoutas

**Affiliations:** 1 University of Crete, Chania, Crete Region, Greece

The article is a comprehensive review of current literature in the fields of clinical psychology and neuropsychology of psychosis particularly bipolar disorder. It comprises several key areas of study in bipolar disorder, describing the patterns of each one: memory, executive functions, attention, and language.

 The article's language is accessible and the cognitive parameters are described analytically. The article consists of extended literature on the topic.

Is the review written in accessible language?

Yes

Are all factual statements correct and adequately supported by citations?

Yes

Are the conclusions drawn appropriate in the context of the current research literature?

Yes

Is the topic of the review discussed comprehensively in the context of the current literature?

Yes

Reviewer Expertise:

Clinical Psychology, Education, Clinical Interventions, School Psychology, Psychotherapy, Counseling

I confirm that I have read this submission and believe that I have an appropriate level of expertise to confirm that it is of an acceptable scientific standard.

# Reviewer report: Reviewer response for version 1

**DOI:** 10.5256/f1000research.155060.r210876

**Published:** 2023-10-12

**Authors:** Andrew Sortwell

**Affiliations:** 1 University of Notre Dame, Notre Dame, Indiana, USA

1. Introduction: The introduction of the research paper offers a clear and engaging overview of neuropsychology, highlighting its focus on understanding the brain-mind-behavior relationship. The emphasis on psychological phenomena such as language, motivation, and memory sets the stage for discussing the relevance of neuropsychology in the context of bipolar disorder.
2. Research Gap: The paper effectively addresses the limited attention given to neuropsychological changes in individuals diagnosed with bipolar disorder compared to other psychiatric conditions like schizophrenia. By identifying this research gap, the authors underscore the importance of studying cognitive abnormalities in bipolar disorder, thus establishing a compelling rationale for the review.
3. Scope and Objectives: The paper defines its scope and objectives succinctly, aiming to compile and analyze existing research on the association between cognitive abnormalities and bipolar disorder. The comprehensive approach, including various stages of the condition and comparative studies with schizophrenia, reflects the thoroughness and relevance of the study.
4. Methodology: Although the paper does not explicitly outline its methodology, it successfully reviews and synthesizes existing research, providing a cohesive analysis of the available literature. The inclusion of diverse studies enhances the credibility of the findings and enriches the discussion.
5. Findings and Analysis: The review delves into the cognitive abnormalities associated with bipolar disorder, exploring research across different stages of the condition. The comparison with schizophrenia adds depth to the analysis, offering a broader perspective on neuropsychological aspects. Additionally, the consideration of psychopharmaceutical interventions contributes to a comprehensive understanding of potential treatment implications.
6. Implications and Future Directions: The authors effectively discuss the etiological implications, therapies, preventions, and prognostic factors related to social competence and quality of life for patients with bipolar disorder. This section offers valuable insights for future research, highlighting the importance of further investigation in this area.
7. Conclusion: The conclusion succinctly summarizes the key findings and emphasizes the critical need for continued research in neuropsychology concerning bipolar disorder. The paper successfully advocates for a heightened focus on cognitive abnormalities, thereby contributing to a deeper understanding of the condition and its potential therapeutic implications.
8. Overall Evaluation: Overall, this research paper offers a comprehensive and insightful review of the association between cognitive abnormalities and bipolar disorder. The study effectively identifies a significant research gap, analyzes existing literature, and provides valuable insights for future research and clinical practice. This contribution is essential for advancing our understanding of the neuropsychological aspects of bipolar disorder and improving the lives of affected individuals.

Is the review written in accessible language?

Yes

Are all factual statements correct and adequately supported by citations?

Yes

Are the conclusions drawn appropriate in the context of the current research literature?

Yes

Is the topic of the review discussed comprehensively in the context of the current literature?

Yes

Reviewer Expertise:

NA

I confirm that I have read this submission and believe that I have an appropriate level of expertise to confirm that it is of an acceptable scientific standard.
